# Initiation and continuation of randomized trials after the publication of a trial stopped early for benefit asking the same study question: STOPIT-3 study design

**DOI:** 10.1186/1745-6215-14-335

**Published:** 2013-10-16

**Authors:** Gabriela J Prutsky, Juan Pablo Domecq, Patricia J Erwin, Matthias Briel, Victor M Montori, Elie A Akl, Joerg J Meerpohl, Dirk Bassler, Stefan Schandelmaier, Stephen D Walter, Qi Zhou, Pablo Alonso Coello, Lorenzo Moja, Martin Walter, Kristian Thorlund, Paul Glasziou, Regina Kunz, Ignacio Ferreira-Gonzalez, Jason Busse, Xin Sun, Annette Kristiansen, Benjamin Kasenda, Osama Qasim-Agha, Gennaro Pagano, Hector Pardo-Hernandez, Gerard Urrutia, Mohammad Hassan Murad, Gordon Guyatt

**Affiliations:** 1 Knowledge and Evaluation Research Unit, Mayo Clinic, Rochester, MN 55905, USA; 2 Unidad de Conocimiento y Evidencia, Universidad Peruana Cayetano Heredia, Lima, Peru; 3 Department of Pediatrics, Children’s Hospital of Michigan, Wayne State University School of Medicine/Detroit Medical Center, Detroit, MI 48201, USA; 4 Department of Internal Medicine, Henry Ford Hospital, Detroit, MI 48202, USA; 5 Basel Institute for Clinical Epidemiology and Biostatistics, University Hospital Basel, Basel, Switzerland; 6 Department of Clinical Epidemiology and Biostatistics, McMaster University, Hamilton, ON L8S 4L8, Canada; 7 Division of Endocrinology, Diabetes, Metabolism, Nutrition, Mayo Clinic, Rochester, MN 55905, USA; 8 Department of Medicine, State University of New York at Buffalo, Buffalo, NY 14214, USA; 9 German Cochrane Centre, Institute of Medical Biometry and Medical Informatics, University Medical Center Freiburg, Berliner Allee 29, 79110, Freiburg, Germany; 10 Center for Pediatric Clinical Studies, University Children’s Hospital Tuebingen, Tuebingen, Germany; 11 Department of Neonatology, University Children’s Hospital Tuebingen, Tuebingen, Germany; 12 Academy of Swiss Insurance Medicine, University Hospital Basel, Basel, Switzerland; 13 Iberoamerican Cochrane Center, CIBER de Epidemiología y Salud Pública, IIB, Sant Pau, 08041, Barcelona, Spain; 14 Epidemiology and Public Health CIBER (CIBERESP), Hospital de la Sant Pau Creu i, Sant Pau, 08041, Barcelona, Spain; 15 Department of Biomedical Sciences for Health, University of Milan, Milan, Italy; 16 IRCCS Galeazzi Orthopedic Institute, Milan, Italy; 17 Department of Nuclear Medicine, University Hospital Bern, Bern, Switzerland; 18 Department of Primary Health Care, University of Oxford, Oxford, UK; 19 Centre for Evidence-Based Medicine, University of Oxford, Oxford, UK; 20 Epidemiology Unit, Department of Cardiology, Vall d’Hebron Hospital and CIBER de Epidemiología y Salud Publica (CIBERESP), Barcelona, Spain; 21 Department of Anesthesia, McMaster University, Hamilton ON L8S 4L8, Canada; 22 Center for Health Research, Kaiser Permanente Northwest, Portland OR 97227, USA; 23 Department of Translational Medical Sciences, Federico II University of Naples, Via Pansini 5, 80131, Naples, Italy; 24 Division of Preventive, Occupational and Aerospace Medicine, Mayo Clinic, Rochester, MN 55905, USA; 25 Norwegian Knowledge Centre for the Health Services, Central Invoicing DFO, PO 4104, Hamar, 2307, Norway

**Keywords:** Randomized controlled trials stopped early for benefit, RCT, Systematic review, Protocol

## Abstract

**Background:**

Randomized control trials (RCTs) stopped early for benefit (truncated RCTs) are increasingly common and, on average, overestimate the relative magnitude of benefit by approximately 30%. Investigators stop trials early when they consider it is no longer ethical to enroll patients in a control group. The goal of this systematic review is to determine how investigators of ongoing or planned RCTs respond to the publication of a truncated RCT addressing a similar question.

**Methods/design:**

We will conduct systematic reviews to update the searches of 210 truncated RCTs to identify similar trials ongoing at the time of publication, or started subsequently, to the truncated trials ('subsequent RCTs’). Reviewers will determine in duplicate the similarity between the truncated and subsequent trials. We will analyze the epidemiology, distribution, and predictors of subsequent RCTs. We will also contact authors of subsequent trials to determine reasons for beginning, continuing, or prematurely discontinuing their own trials, and the extent to which they rely on the estimates from truncated trials.

**Discussion:**

To the extent that investigators begin or continue subsequent trials they implicitly disagree with the decision to stop the truncated RCT because of an ethical mandate to administer the experimental treatment. The results of this study will help guide future decisions about when to stop RCTs early for benefit.

## Background

The decision of whether to stop a randomized control trial (RCT) for apparent benefit before its planned completion is complex and requires consideration of ethical, statistical, and practical issues [1]. The main rationale for stopping is to avoid denying current and future control group participants a beneficial treatment, and to ensure rapid dissemination of that treatment [2]. A correct decision requires the wise judgment of the study investigators and, preferably, of an independent data monitoring committee (DMC) typically including trialists with both clinical and statistical expertise [3]. The DMC needs to attend to the interests of future patients and society at large, while considering the impact of their results on the wider community of clinicians, researchers, and evidence users [2]. Considerations include the risk of disseminating an overestimation of the treatment effect on the primary outcome and ensuring that the optimal information regarding toxicity and secondary outcomes is also captured, particularly if adverse events occur late in the course of the trial [4].

A systematic review (Study of Trial Policy of Interim Truncation-1 (STOPIT-1)) developed by our group to evaluate the epidemiology and reporting quality of RCTs stopped early for benefit (truncated RCTs (tRCTs)) found that this type of trial was becoming more common, and often failed to adequately report relevant information about the decision to stop early [5]. A subsequent systematic review (STOPIT-2) compared 91 tRCTs asking 63 different research questions to 424 non-tRCTs asking similar questions. tRCTs tended to overestimate the magnitude of benefit by one third (average), regardless of the use of statistical stopping rules. Moreover, almost two thirds of the pooled effects of the non-tRCTs failed to demonstrate benefit [6].

These results raise serious concerns about the potential impact of stopping trials early on the body of evidence and therefore on current health policies. First, investigators have disseminated an estimate of effect that is, on average, substantially overestimated; how stakeholders best incorporate this evidence in generating subsequent best estimates of effect is uncertain. Second, this represents a lost opportunity to generate more precise and higher quality evidence about benefits and harms. Third, the publication of a trial stopped early for benefit may stop further research addressing the same question, which may distort the effect size (overestimating the effect and making it more imprecise) of the whole body of evidence [7].

There are a number of possible reasons investigators launch a new study with a similar research question to that of a tRCT, or continue one in progress (we shall refer to trials continued or started despite a previous tRCT as 'subsequent RCTs’ (sRCTs). First, they may be unaware of the existence of the tRCTs. This seems unlikely, given that researchers are usually aware of ongoing research in their field, and that tRCTs are commonly published in high impact journals, and often receive appreciable media attention [5,6]. A second possibility is that investigators are aware of the findings of the tRCT but remain unconvinced of the observed large benefit (even of whether there is any important benefit) and thus deem it ethical to allocate patients to not receive the new treatment. The decision to continue a current trial or start a new one answering a similar research question may represent an implicit different judgment with the previous decision to stop early and to the body of evidence in general.

If sRCTs are common, it suggests that current practice of stopping RCTs for apparent benefit might not be acceptable by many other scientists and not sufficiently conservative either by reaching different conclusions than the body of evidence or showing highly unreliable estimates. Understanding the decisions to initiate, continue, or begin sRCTs is likely to provide insights that ultimately improve the policies and procedures of clinical trial oversight and the credibility of clinical research.

### Objective

The present study aims to examine how often randomized trials are launched or continued after the publication of a tRCT asking the same or sufficiently similar research question. We will also analyze trials that were stopped in response to such external evidence. Our study involved two primary research questions. First, what is the proportion of tRCTs that are followed by a sRCT addressing the same or closely similar research question? Second, what factors/rationale are associated with the launching or continuation of a sRCT after the publication of a tRCT?

## Methods/design

### Overview of methods

**Figure 1:**
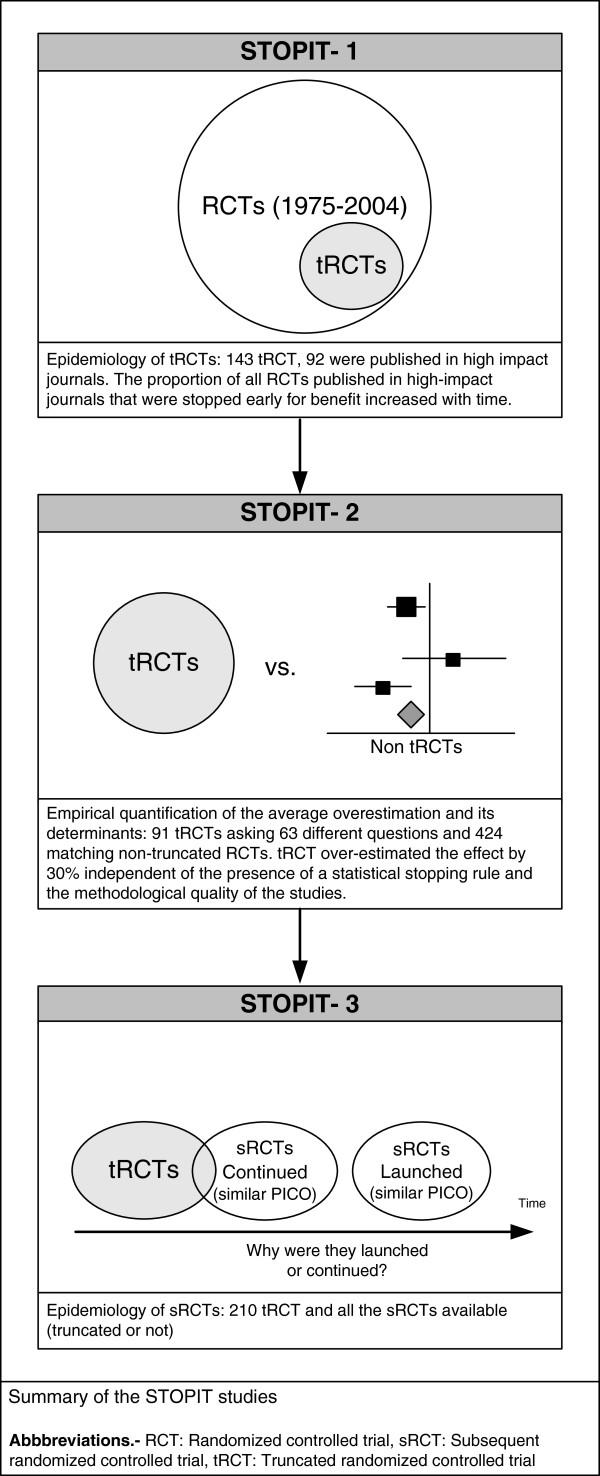
Summary of the STOPIT studies.

The design and findings of STOPIT-1, 2, and 3 are depicted in Figure 1. STOPIT-1 included 143 tRCTs. During the development of STOPIT-2, researchers identified 14 additional tRCTs through hand search and personal contact. Recently, the search strategy for tRCTs was updated yielding a total of 210 tRCTs.

In STOPIT-3, we will search for and analyze RCTs (truncated or not sRCTs) published after the publication of each primary tRCTs. If we have a tRCT with sRCT that is also a tRCT, each study will be analyzed independently and two search strategies will be developed. This means that we will include this tRCT as a sRCT for the first tRCT, but it will be also analyzed as a tRCT itself.

**Figure 2:**
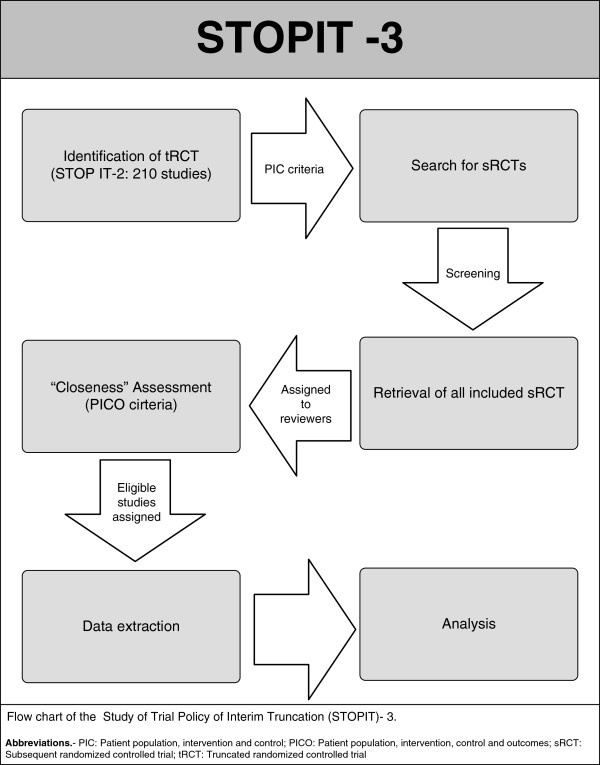
Flow chart of the STOPIT-3.

Following the same approach used in STOPIT-1, we will determine the prevalence of tRCTs reporting having stopped early for benefit. The methodology of this present study is summarized in Figure 2.

### Literature search

With input from study investigators (MB, GJP, JPD, MHM) experienced in conducting systematic reviews, a reference librarian (PJE) will design and execute individual search strategies for each tRCT included in the database. These electronic search strategies will use controlled vocabulary and text words taking into account the characteristics of the population involved, the intervention, and comparison used. We will not consider the outcomes assessed in the tRCTs as a part of the search strategy to ensure high search sensitivity. For each tRCT, we will search the electronic databases (Ovid MEDLINE, Ovid Embase, Ovid Cochrane Library, Web of Science, Scopus, and PsycINFO) from the stated date of publication of the tRCTs through to the present time.

### Eligibility criteria

In order to consider a trial as an eligible sRCT it has to be either: 1) launched (that is, started enrollment) after the publication date of a matching tRCT; sRCT may, or may not, be terminated prematurely for benefit based on its own data; or 2) ongoing at the time of publication of the tRCT, and achieving the calculated sample size and planned study duration after the tRCT publication date. It does not matter whether the sRCT was stopped early or not.

**Figure 3:**
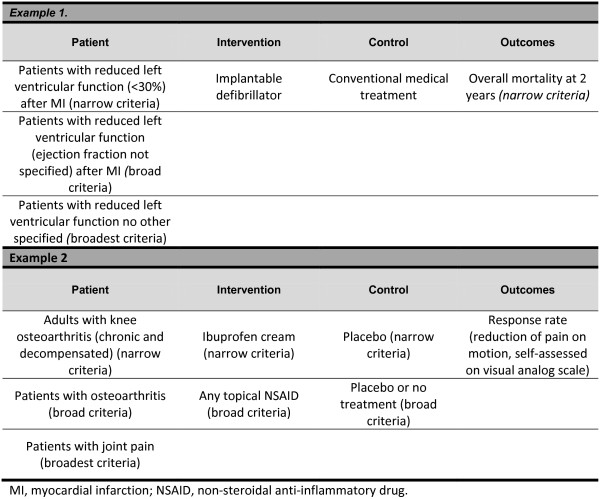
Closeness assessment.

As a general approach we will consider trials as sRCTs of a tRCT if pooling them together in meta-analysis seems adequate. An example is shown in Figure 3.

### Study selection

We will use an online reference management system (DistillerSR, Ottawa, ON, Canada) for study selection. Reviewers will first calibrate their judgments using a small set of reports. Two reviewers will independently screen for potential eligibility the title and abstract of each citation that result from the search strategy. We will obtain the full text version of any citation that either reviewer deems potentially eligible.

Two reviewers will independently evaluate each full text for eligibility. We will measure agreement using the kappa or phi statistics, as appropriate (the latter is appropriate when the distribution of agreement is extreme). This will be provided in real-time by the online system. Disagreements will be resolved by discussion or, if necessary, by third party adjudication. For all eligible sRCTs, during data extraction, reviewers will judge whether the patients, interventions, comparators, and outcomes match those of the corresponding tRCT as narrow (very closely matched), broad (closely matched), or broadest (matched, but appreciable differences). For this purpose we will use the criteria previously established in STOPIT-2.

Thus, we will classify each element of the sRCT (patients, interventions, comparators, and outcomes; for the assessment of outcomes we will consider the outcome used for stopping the tRCT) as very close (termed as 'fits the narrow criteria’), moderately close (termed as 'fits the broad criteria’), and less close (termed as 'fits the broadest criteria’) relative to the tRCT elements. We will also check the level of agreement (kappa or phi statistics) for this judgment. Disagreements in relation to similarity of one level or greater will require adjudication by a third reviewer [6].

Based on these criteria we will classify each sRCT relative to its tRCT as: more or less identical; similar, but not identical; and broadly similar.

**Figure 4:**
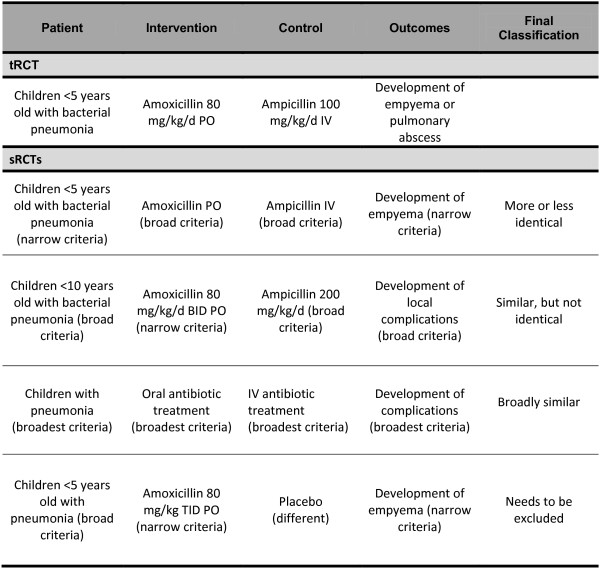
Final closeness assessment.

This will be a subjective process done in duplicates. Disagreements will be resolved by consensus or, if necessary, third party arbitration (Figure 4).

### Data collection and extraction

We will collect data in pre-piloted standardized electronic forms designed using the online reference management system (DistillerSR). Two reviewers will independently extract the information from each report. We selected these variables (Additional file 1: Table S1) based on possible hypothesized explanations of why sRCT investigators decided to continue or not with the conduct of their trial, or launched it despite the presence of a tRCT.

### Outcomes of interest and statistical analysis

The main outcome of interest is the proportion and associated 95% confidence interval (CI) of tRCTs that had at least one sRCT.

In multivariable regression, we will address the possible associations between the presence or absence of a sRCT (dependent variable) and the characteristics of the tRCTs (independent variables) including: risk of bias, magnitude of effect, number of events, funding, presence of DMC, explicit use of a stopping rule, and impact factor of journal of publication. The rationale for these variables is that increased risk of bias, small number of events, conflicts of interest, lack of DMC or stopping rule, and low journal impact factors are all possible reasons for sRCT investigators to be less convinced of the tRCT results. We will adjust the analysis by the year of tRCT publication (more recent tRCTs are expected to have a lower number of sRCTs, since there would not be sufficient time for sRCTs to accrue, and previous publications regarding the dangers of early stopping may have decreased the number of tRCTs in which early stopping is at highest risk of misleading overestimates).

### Risk of bias assessment

The risk of bias of most of the included tRCTs was assessed during the development of the previous studies using the items of the Cochrane risk of bias tool [8]: allocation concealment (documented as central independent randomization facility or numbered/coded medication containers prepared and distributed by an independent facility (for example, pharmacy)); blinding of participants, care providers, and outcome adjudicators (blinding of participants and care providers will be rated as 'probably yes’ when trial report states 'double blinded’ or 'placebo controlled’); loss to follow-up (difference between the number of participants randomized and the number of participants with data for the outcome of interest), and adherence to the intention-to-treat principle. We will follow the same approach for any new or not assessed tRCT.

### Optimal information size and the progression of the body of evidence

We will calculate for each research question (tRCT and its sRCT) the optimal information size (relative risk reduction (RRR) 25%, weighted mean control group event rate, α = 0.05, β = 0.90) and Lan-DeMets sequential monitoring boundaries at the time of publication of the tRCT using all relevant trials published to that point. We will also calculate the necessary information size to obtain conclusive evidence of a plausible RRR of 25%, that is, the difference between the optimal information size and the actually available information size at the time of publication of the tRCT. We will check how close the boundary of the 95% CI of the summary effect at the time of the tRCT publication is to no effect (that is, 1 since we will be dealing only with relative risks). Lastly, we will determine if there is an association between the number of events and chances of the existence of sRCTs.

In addition, we will estimate the prevalence of trials stopped early for benefit using the newly found sRCTs (after STOPIT-1) that were stopped early as the numerator, and all RCTs published in all MEDLINE-indexed journals or the subset of RCTs published in high impact journals as the denominator. Therefore, we will be able to compare the prevalence of stopping early before and after the publication of STOPIT-1 and 2, and determine if these publications had an impact on trial conduct.

### Sensitivity analysis

We plan to conduct two sensitivity analyses. The first one will be based on the closeness of the sRCTs to the research question of the tRCTs (more or less identical; similar, but not identical; and broadly similar). Considering that there is no objective method to assess closeness of research questions it is plausible that investigators of sRCTs would continue their trial if their question were less close to the tRCT. We will test the impact of our assumptions it the results using a sensitivity analysis including only the sRCT-tRCT pairs with clear identical research questions according to the closeness classification given to each sRCT during the study selection phase.

The second sensitivity analysis will compare two definitions of sRCTs based on the timeframes of 6 and 12 months between the publication of the tRCT and the launching of the sRCT. The rationale for this analysis is that at least 6 to 12 months are needed to assure that a sRCT is truly a subsequent trial and not a parallel trial, which would make it impossible for the investigators to be aware of the tRCT.

### Author contact

We will contact the principal investigators of the sRCTs to further explore the reasons why they launched or continued trials after the tRCT. We will use a short, standardized web-based survey instrument developed with SurveyMonkey (Palo Alto, CA, USA) (Additional file 2: Table S2). We will make two attempts by email per contact author at a 2-week interval. If the author’s email address is not available we will make contact by mail or telephone. In an effort to increase our response rate we will also try to contact both the first and senior author following the same approach. If after these attempts we do not receive an answer we will try to contact them (contact, first, and senior authors) by telephone. As a last resource, we will try to contact the other authors listed in each publication following the same approach.

### Reporting

We will report this study in accordance with the recommendations set forth by the Preferred Reporting Items for Systematic Reviews and Meta-Analyses (PRISMA) workgroup [9], and the recommendations developed by the International Society for Pharmacoeconomics and Outcomes Research (ISPOR) [10]. We will present evidence tables for each tRCT including description of the population characteristics, interventions, methodological quality, and main findings.

## Discussion

STOPIT-1 and 2 described the epidemiology of trials stopped early for benefit, including how often early stopping occurs, average overestimation of effect estimates, and its predictors (truncated RCTs having fewer than 500 events). In this systematic review, we will try to measure the impact of a tRCT on the body of evidence, and determine specifically the frequency of sRCTs and determinants of their occurrence. We will try to find the ideal information size for these trials.

### Strengths and limitations of our protocol

The strengths of this study include a systematic and extensive literature search for each of the included tRCTs performed by an experienced librarian and supported by methodological experts. We will also contact authors of sRCTs, and obtain their feedback and rationale. Two reviewers with experience in the development of systematic reviews will assess each study independently, thus decreasing the risk of error and strengthening confidence in the selection process. We have a priori developed this study protocol and included sensitivity analyses to determine if the assumptions we made may affect study conclusions to a certain extension.

Limitations include our reliance on published and indexed publications to identify sRCTs. Considering that the previous publication of a tRCT may reduce the chances of a sRCT to be submitted and eventually published, we are facing a new potential factor for publication bias. In addition, a known limitation of STOP-IT 1, 2, and 3 is the inaccuracy of identifying tRCTs, since truncation is frequently not clearly described in the abstracts or full reports of RCTs [11].

### Ethical and data monitoring implications

The results of this systematic review will impact on the development of future trials and the decision-making process of DMCs. The ultimate goal of a DMC should be to share benefits of an effective treatment as soon as there is sufficient confidence in the magnitude of its effects, both benefits and harms [1]. Sufficient confidence would translate into an ethical mandate to offer the intervention to all patients, and to no longer randomize patients to the possibility of not receiving the intervention.

For each sRCT which their authors knew about the existence of previous tRCT on the same research question, we can infer that authors agreed that randomizing patients continues to be ethical. In such instances, we can conclude that the stopping rule used had serious limitations. If a large proportion of tRCTs are associated with sRCTs this would mandate a re-evaluation of the criteria currently used to judge the appropriateness of stopping early for benefit.

### Public engagement in science

The results of this review will impact on several aspects of public interest. Many different interests converge into releasing the results earlier of a study showing significant benefits. Institutional review boards and funding agencies take into account previous research made in an area before approving a new trial. Following the same approach, journals should consider previous publications before accepting a new trial. Therefore, the publication of a tRCT may slow or prevent future trials answering the same or a similar question, generating a 'freezing effect’. It is possible that our study will identify such a freezing effect if we find an old tRCT that was not followed by any sRCTs and the body of evidence remained inconclusive after the publication of the tRCT [12]. Inference regarding a freezing effect involves the assumption that trial publication, continuation, or initiation is driven by the conclusiveness of the body of evidence and ignores other factors that relate to funding availability, economy, and other temporal trends.

tRCTs are published in higher impact journals and gain more media attention; therefore, the media and the public may be impacted more by their results, and they may have increased confidence in the findings. However, if we find a large number of sRCTs, this may suggest that the academic community did not accept the ethical judgment of trials DMCs and trialists. This would mandate a re-evaluation of the current stopping criteria and the confidence the public should have about the findings.

## Conclusion

Given the increasing frequency of tRCTs published in high impact journals and the fact that these studies may overestimate the real effect of an intervention, it is important to evaluate the impact of tRCTs on the body of evidence. Distortion of the body of evidence can subsequently lead to biased systematic reviews, and impact guideline recommendations and patient care.

## Trial status

The systematic review is currently in full text screening phase. Completion is expected by November 2013.

## Abbreviations

CI: Confidence interval; DMC: Data monitoring committee; ISPOR: International Society for Pharmacoeconomics and Outcomes Research; PRISMA: Preferred Reporting Items for Systematic Reviews and Meta-Analyses; RCT: Randomized control trial; RRR: Relative risk reduction; sRCT: Subsequent randomized control trial; STOPIT: Study of Trial Policy of Interim Truncation; tRCT: Truncated randomized control trial.

## Competing interests

The authors declare they have no competing interests.

## Authors’ contributions

GJP has been involved in the organization of the project since the beginning (design of search strategies, retrieving of studies, study selection, and data extraction), wrote the first version of the manuscript, and was in charge of its development. JPD has been involved in the organization of the project since the beginning (design of search strategies, retrieving of studies, study selections, and data extraction), collaborated with the first version of the protocol, reviewed it, and approved it before submission. PJE designed the search strategies for the project and reviewed the protocol. MB conceptualized the study, supervised all the process, and reviewed the manuscript before submission. VMM collaborated with the first version of the protocol, reviewed it, and approved it before submission. EAK, JJM, DB, SS, SDW, QZ, PAC, LM, MW, KT, PG, RK, IFG, JB, XS, AK, BK, OQA, GP, HP, and GU are reviewers and reviewed and provided feedback to the manuscript before submission. MHM participated in the design of the study, performed the statistical analysis, and reviewed and approved the manuscript before submission. GG participated in the design of the study, performed the statistical analysis, and reviewed and approved the manuscript before submission. All authors read and approved the final manuscript.

## Supplementary Material

Additional file 1: Table S1 Variables to be extracted. Click here for file

Additional file 2: Table S2 Form for contacting the authors of sRCTs. Click here for file
