# Supplementary material for: Initiation and continuation of randomized trials after the publication of a trial stopped early for benefit asking the same study question: STOPIT-3 study design
Source: Trials. 2013 Oct 16;14:335. doi: 10.1186/1745-6215-14-335 (PMC3874848; doi:10.1186/1745-6215-14-335)
Supplement: Additional file 1: Table S1 — Variables to be extracted. [file 1745-6215-14-335-S1.doc]

**Additional file 1: Table S1.** Variables to be extracted

| **Variable** |  | | **Data** |
| --- | --- | --- | --- |
| **tRCT** | | | |
| Publication date |  | Month and year | |
| Existence of a DMC |  | Yes | |
|  |  | No | |
|  |  | Not mentioned | |
| Pre-planned stopping rule |  | Yes (which one) | |
|  |  | No | |
|  |  | Not mentioned | |
| Magnitude of the effect |  |  | |
| Magnitude of effect in the pooled estimate at the time of publication |  |  | |
| Total number of events |  |  | |
| Total number of events in the index tRCT + number of events in all RCTs published before the tRCT (meta-analysis) |  |  | |
| Prestige of journal in which the tRCT was published |  | Top ranked peer reviewed journals: *NEJM*, *Lancet*, *JAMA*, Ann Intern Med, *BMJ* | |
|  |  | Impact factor as continuous independent variable (surrogate for prestige) | |
| Funding |  | Industry/public | |
| sRCTs | | | |
| Publication date |  | Month/year | |
| Recruitment period (date) |  | Month/year to month/year | |
| Follow-up period (date) |  | Follow-up period (date) | |
| Where was this study developed? |  |  | |
| At the time of the publication of the tRCT this sRCT was: |  | Not launched yet | |
|  |  | Patient recruitment phase | |
|  |  | Follow up phase | |
|  |  | Unclear | |
| Closeness of a successive trial to the tRCT |  | Population (very close/moderately close/less close) | |
|  |  | Treatment arm (very close/moderately close/less close) | |
|  |  | Control arm (very close/moderately close/less close) | |
|  |  | Outcome (very close/moderately close/less close) | |
| Does this trial cite the tRCT in its report(s)?^a^ |  | Yes/no | |
| Is there an explicit rationale to continue or launch the trial despite the positive evidence of effect from the tRCT? |  | Problems in directness: | |
|  |  | a) Different population (for example, subgroups) | |
|  |  | b) Intervention | |
|  |  | c) Outcome measured (for example, follow-up not long enough) | |
|  |  | d) Other reason not listed | |
|  |  | Do not believe the answer: | |
|  |  | a) Insufficient number of patient events | |
|  |  | b) Not all previous trials showed significant positive results | |
|  |  | c) Confidence intervals too wide | |
|  |  | d) High risk of bias | |
|  |  | e) Other reason not listed | |
|  |  | Need for more data about possible harms | |
|  |  | Need for more data on other clinical outcomes | |
|  |  | Other | |
| Existence of a DMC |  | Yes/no/not mentioned | |
| Did investigators break the randomization code and conduct an interim analysis, or did they look at the available external data? (If they ran a meta-analysis including their own and previous trials.) |  | Yes/no/not mentioned | |
| Did the authors consider results from the tRCT in their sample size calculation? |  | Yes/no/not mentioned | |
| Results of the new trial on the variable for which the previous study stopped early |  | Magnitude of effect | |
|  |  | *P* value | |
| Does this sRCT call for more studies? |  | Yes/no/not mentioned | |
| Was this study stopped due to publication of results of tRCT? |  | Yes/no | |

^a^Clear indication of the awareness of investigators about the tRCT. DMC, data monitoring committee; RCT, randomized control trial; sRCT, subsequent randomized control trial; tRCT, truncated randomized control trial.
