# Supplementary material for: Initiation and continuation of randomized trials after the publication of a trial stopped early for benefit asking the same study question: STOPIT-3 study design
Source: Trials. 2013 Oct 16;14:335. doi: 10.1186/1745-6215-14-335 (PMC3874848; doi:10.1186/1745-6215-14-335)
Supplement: Additional file 2: Table S2 — Form for contacting the authors of sRCTs. [file 1745-6215-14-335-S2.docx]

**Additional file 2: Table S2**. Form for contacting the authors of sRCTs

| **Contacting the authors of sRCTs** | | | | | | |
| --- | --- | --- | --- | --- | --- | --- |
| 1 | Were you aware of the tRCT when you launched/ continued your trial? | Yes | Did you consult your DMC at the time you learned of the tRCT results? | Yes | | |
|  |  |  |  | No | | |
|  |  |  | Did you consider stopping your trial at that point? | Yes | Why? | The magnitude of the effect in the tRCT |
|  |  |  |  |  |  | My own data (interim analysis) |
|  |  |  |  |  |  | IRB asked me to do it |
|  |  |  |  |  |  | DMC asked me to do it |
|  |  |  |  |  |  | Other. Please explain. |
|  |  |  |  | No |  | |
|  |  | No | Did you become aware of the tRCT after launching/completing your trial? | Yes | | |
|  |  |  |  | No | | |
| 2 | To which extent did you think that your trial addressed the same question as the tRCT? | Very close |  | | | |
|  |  | Moderately close |  | | | |
|  |  | Less close |  | | | |
|  |  | Not close at all |  | | | |
| 3 | Would you think that the ethics committee approving the trial was aware of the tRCT? | Yes |  | | | |
|  |  | No |  | | | |
|  |  | I do not know |  | | | |
| 4 | Would you think that the sponsor of your trial was aware of the tRCT? | Yes |  | | | |
|  |  | No |  | | | |
|  |  | I do not know |  | | | |
| 5 | If you were aware of the tRCT and still launched your trial or became aware of it and did not halt you’re your trial, what were your reasons of doing so? | Did not believe the results of the tRCT | Insufficient number of events | | | |
|  |  |  | Potential bias with study being stopped early | | | |
|  |  |  | Not all previous trials showed significant positive effects | | | |
|  |  |  | Confidence intervals too wide | | | |
|  |  |  | Other. Please explain | | | |
|  |  | My trial was different | Different patient population (e.g. subgroups intervention) outcome, e.g.) | | | |
|  |  |  | Different intervention | | | |
|  |  |  | Different control arm | | | |
|  |  |  | Different outcomes (e.g. Harm outcomes) | | | |
|  |  |  | Longer follow up | | | |
|  |  |  | Other. Please explain | | | |
|  |  | Other | Please explain. | | | |
| 7 | Did you use estimates from the tRCT for your own sample size considerations? | Yes |  | | | |
|  |  | No |  | | | |
| 8 | Did you conduct an interim analysis | Yes | Why? What did it say? Please explain. | | | |
|  |  | No | Why? | | | |
| 6 | Are you aware of other trials that were planned but not launched or stopped because of the tRCT publication? | Yes | How many were not launched? | | | |
|  |  |  | How many were stopped? | | | |
|  |  | No |  | | | |
| ***Abbreviations.-*** *sRCTs: subsequent randomized controlled trials, tRCT: truncated randomized controlled trial* | | | | | | |
